# Changes in the Rhythm of Speech Difference between People with Nondegenerative Mild Cognitive Impairment and with Preclinical Dementia

**DOI:** 10.1155/2020/4683573

**Published:** 2020-04-14

**Authors:** Juan J. G. Meilán, Francisco Martínez-Sánchez, Israel Martínez-Nicolás, Thide E. Llorente, Juan Carro

**Affiliations:** ^1^Faculty of Psychology, University of Salamanca, Salamanca, Spain; ^2^Institute of Neurosciences of Castile and Leon, Salamanca., Spain; ^3^Faculty of Psychology, University of Murcia, Murcia, Spain

## Abstract

This study explores several speech parameters related to mild cognitive impairment, as well as those that might be flagging the presence of an underlying neurodegenerative process. Speech is an excellent biomarker because it is not invasive and, what is more, its analysis is rapid and economical. Our aim has been to ascertain whether the typical speech patterns of people with Alzheimer's disease are also present during the disorder's preclinical stages. To do so, we shall be using a task that involves reading out aloud. This is followed by an analysis of the recordings, looking for the possible parameters differentiating between those older people with MCI and a high probability of developing dementia and those with MCI that will not do so. We found that the disease's most differentiating parameters prior to its onset involve changes in speech duration and an alteration in rhythm rate and intensity. These parameters seem to be related to the first difficulties in lexical access among older people with AD.

## 1. Introduction

Mild cognitive impairment (MCI) is a nondegenerative heterogeneous prodrome involving mild cognitive declines in short-term memory processes, as well as deficits in attention or in access to words. The changes are bigger than are to be expected in people of a certain age and with a given level of education, but they do not affect or interfere with their everyday lives [1]. MCI tend to go undiagnosed, given the financial and personal cost of conducting biomarker tests. Furthermore, such declines are typically attributed to normal aging, even though some of them may be masking a preclinical stage of Alzheimer's disease (AD) rather than an MCI. This fact tends to be ignored, as only 15-20% of people with MCI subsequently develop AD [2]. Their early diagnosis is important because it will enable families to plan their future, involve patients in the control of risk factors, promote research into the disease, provide the appropriate care, foresee new symptoms, and arrange treatment that is more effective the sooner it is given.

Researchers' overriding goal today regarding dementia is to perfect the early screening of AD [3], distinguishing between older people with the typical deficits of MCI, but who do not have AD, from those that are indeed developing the disease. To do so, we focus on other cognitive processes that are more likely to reveal preclinical deficits, such as verbal changes in speech related to language production, especially impairments in lexical and semantic access [4]. Weiner et al. [5] have found a close correlation between the severity of dementia and different linguistic metrics; nevertheless, the results in studies with patients in the mild-to-moderate stages of AD [6, 7] cannot be generalized to earlier stages of the disease when compensatory abilities may result in more subtle impairments in functional language [8]. However, specific studies involving people with MCI have identified subtle language deficits [9] in tasks involving the naming of images and semantic tests, which are usually reflected in problems finding the right word in a given situation [10], changes in the frequency of words used for more common words [11], temporal changes in spontaneous language [3] with deficits in verbal fluency tasks [12], and phonemic paraphasia due to the difficulty in producing speech sounds in the right order [13].

This means that individuals with MCI would have differences in their general pattern of discourse, rather than differences in specific speech parameters [14]. Spontaneous speech production involves the use and coordination of multiple cognitive and psychological processes that include recovering from semantic memory, paying attention to speech, planning phonological and articulatory processes, and planning syntax [15]. Researchers are trying to identify the speech markers that distinguish between those individuals with MCI that will subsequently develop AD and those who will not [16]. These microlinguistic changes seem to be associated with pathologies unrelated to memory but instead to syntactic and phonological linguistic processes similar to those manifested by logopenic progressive primary aphasia (logopenic PPA or LPA, [17]). People with LPA are characterized by less dense and inaccurate speech planning [7], with difficulty in finding words, simplified syntaxis and semantics, paraphasia, and circumlocutions, with all the symptoms also appearing in the early stages of AD [18].

Several studies have recently sought to identify MCI with technical advances in speech analysis [19]. The coordination functions will have a subtle impact on the rhythm of speech. Speech in people with MCI is characterized by a longer speech time due to the presence of stammers and articulatory disfluencies that may even interrupt speech and to longer hesitations [20]. There are also longer silent pauses (as an absence of speech without vocalization) and a lower enunciation rate [21], both in speech rate with hesitations and in articulation rate without hesitations [22]. Some authors proposed the study of the flow of speech into an especially sensitive neuropsychological method for investigating cognitive processes such as speech production and planning [23]. The changes in rhythm will affect the systematic organization of speech units in time [24]: (1) duration, (2) syllabic intervals, (3) fundamental frequency and spectral analysis, and (4) intensity:

1. Duration: different studies contend that the subtle impairments in speech duration are an indication of changes in the temporal functions of phonation [25]. Individuals with MCI record longer speech duration; a lower speech rate, as well as more and longer pauses [22]; or an inappropriate temporal distribution of pauses [26]. Seminal studies have argued that the production of a sentence directly reflects the utterance's syntactic structure or its syntactic complexity [27, 28]. Lehiste [28] identifies the duration of phonation as the clearest clue to the way of unravelling syntactical structures based on their relationships: lengthening of syntactic boundaries and increase in pauses—in both number and length unravelling especially between the boundaries of each syntactic segment
2. Syllabic intervals: Cera et al. [14] argue that these disorders are related to phonetic-motor planning, which leads to poor pronunciation and an alteration in phonological planning and syllabic rhythm. Rhythm has been defined as an effect involving the isochronous recurrence of some type of speech unit [29]. Although it differs across languages (Spanish is considered a good example of syllable timing), there are comparable parameters such as articulation rate (measure of rate of speaking in which all pauses are excluded from the calculation), speech rhythm (which includes four temporal variables for the signaling of rhythmicity: fundamental frequency, syllabic duration, syllabic energy, and spectral dynamics), and speech ratio (ratio of the time spent in vocalizing—speech time—to total time—speech time plus pause time—involved in the speech), which are called fluency
3. Frequency: other studies have contended that the impairment is related to problems in phonological planning [30] due to changes in the control of vocal execution, which has a negative influence on the point, mode, and tension of articulation. An utterance's tonal curve may record deviations of the tone in the final prosodic boundaries or in the continuation of another utterance [31, 32]. There may also be deviations in the scaling of pitch accents within an utterance, with the preceding alterations influencing the development of the tonal sequence [33]. Fraser et al. [7] have posited that changes in the spectral features of sound would be the characteristic ones at the initial onset of AD, as this would affect the special centroid parameters such as mel-frequency cepstral coefficients (MFCCs), the spectral energy, flux, variance, skewness, kurtosis, and slope
4. Intensity: finally, we may also expect changes in the parameters of intensity due to problems in the articulatory and prosodic sequence. The interdependence between phonatory and articulatory aspects has been repeatedly verified [34]. These aspects appear to be especially important during reading. In fact, authors such as Fodor [35] add the function of an implicit fluency present in reading that permits a reanalysis of a text's semantic and syntactic structure or the groupings of each language's characteristic words into complex sentences

Our aim here is to investigate these four aspects (speech duration, syllabic rate, spectral analysis, and the control of vocal intensity) and verify whether these parameters record statistical differences between those individuals with MCI with nondegenerative disorder (nodMCI) and individuals with MCI with preclinical symptoms of dementia and who will probably develop AD (preAD). Speakers use speech fluency based on suprasegmental elements to facilitate word recognition and their syntactic components. As a hypothesis, we consider that the difficulties in these subtle parameters on controlling speech fluency may be clinically useful for revealing the intricacies of speech fluency and rhythmic performance in individuals with early dementia.

In the preview studies, we have obtained a discriminant screening function for the diagnosis of AD based on the Voice Analysis Diagnostic of Alzheimer's Disease (VAD-AD, [36]), which rates the probability that an individual's speech corresponds to someone with nonpathological senescence (NPS) or to someone with AD. The outcome was that the participants were correctly classified in 92.4% of the cases (a classification of normality is predicted in 97% of cases). The variables used in this algorithm—similar parameters to those found by other authors—were age; mean of the voice's minimum amplitude in each utterance (Amplit Min); mean of the value of the maximum amplitude difference value in each utterance (Amplit Difference Max Mean); location of the mean frequency within the interval of frequencies (asymmetry); the value of the standard deviation of the first formant (sdF1); the size of the bandwidth of the third formant (F3 B3); pitch trajectory (sum of absolute intervals) within syllabic nuclei, divided by duration (in ST/s) (Traj Intra); normalized Pairwise Variability Index (nPVI); the standard deviation of the harmonics-to-noise ratio (sdHNR), and the harmonics-to-noise ratio (HNR) extracted from the Acoustic Voice Quality Index (AVQI HNR). The characteristics identify people that have already developed AD.

Nevertheless, now, we use a cohort of individuals with MCI that have not yet developed the disease's clinical symptoms. We believe that, in this sample, we could find new and different variables that show deficits at the beginning of the disease, that is, parameters that differentiate those who have the disease from those who do not. Therefore, we set out to analyze other parameters that could, in theory, be more sensitive in the disease's preclinical stages. The voice and speech parameters evaluated are grouped into various categories: (1) those related to fluency (duration, phonation time) and speech fluency: we analyzed the phoneme's number per second without hesitations (articulation rate), phoneme's number per second with hesitations (speech rate), and number of voice pauses (number of pauses); (2) related to speech rhythm: we analyzed the number of syllabic intervals, average duration of syllabic intervals (Syll_Interv_DAverage), standard deviation of syllabic intervals (Syll_Interv_*Δ*Standar), coefficient of variation in the duration of syllabic intervals (Syll_Interv_DVarco), and nPVI. The nPVI is the mean of the differences in duration between two successive syllabic intervals in speech, divided by the sum of those same intervals; (3) fundamental frequency (F0 mean) and those derived from the spectrum analysis of the amplitude of the voice signal: asymmetry (the center of gravity is at one end of the interval of frequencies or manifests a characteristic skewness), center of gravity (CoG_Hz) indicating the spectral area that concentrates most of the energy and its SD (mean distance from the center of gravity), and those derived from the averaged spectrum analysis of the amplitude of the voice signal, conducting an analysis of the long-term average spectrum. The LTAS represents speech energy across the frequency in decibels (dB) and quantifies the quality of voices flagging differences between age and dysphonic voices. We analyzed the LTAS mean and the LTAS_sd in the spectrum between the following range bands: 50 Hz-1 kHz, 1 kHz-2 kHz, and 2 kHz-4 kHz; (4) energy (mean intensity in dB and standard deviation); and (5) voice quality-related measures as the mean of the fluctuation of tone between successive voice periods (Jitter_loc), the mean fluctuation of the amplitude between successive voice periods (Shimmer Loc_dB), percentage of voice breaks (%Voice break), and the ratio between inharmonic aperiodic voice components and harmonic periodic components (Harmonics-to-Noise ratio_HNR), as well as the Acoustic Voice Quality Index (AVQI) [37] on the connected speech index.

## 2. Material and Methods

### 2.1. Participants

We collected the utterances of 86 individuals with MCI as determined by the dementia screening test (Dem-Detect, [38]) selected from a larger pool of speakers from an existing study. We have conducted three sessions of neuropsychological evaluation with each participant that includes a complete anamnesis, assessment of activities of daily living, and a cognitive and psychological evaluation. Inclusion criteria for the larger study comprised normal or corrected-to-normal vision and hearing, sufficient educational level to perform a reading task (six years of primary education), and being a native speaker of Spanish. The participants recorded the following mean scores: age, 79.36 (SD = 9.58, range: 60-96); years of schooling, 8.71 (SD = 0.20); 23.41 out of 30 (SD = 4.59) in the Mini-Mental State Examination (MMSE, [39]); 4.84 of 8 (SD = 1.98) in the Memory Impairment Scale, a test that involves using free recall and cued recall to retrieve four words [40]; 19 of 30 (SD = 10.59) in the Boston Naming Test (brief version, [41]); and 31.70 words of 40 (SD = 6.92) in the fluency SET Test of semantic categories [42]. All measurements reflect typical MCI scores. An individual was considered to be suffering from MCI when they did not exceed the cutoff point for cognitive normality in at least two of the tests taken. Participants with severe depression (score of >6 on the Goldberg Test) or other serious psychiatric disorders were excluded. Written informed consent was obtained from all the participants.

We classified the participants according to the Voice Analysis Diagnostic of Alzheimer Disease (VAD_AD algorithm, [36]) and conducted tests for comparing the two resulting groups, nodMCI and preAD, on those parameters proposed for distinguishing between the samples (see Table 1). Based on the final score, the procedure provides the degree of probability of belonging to the NPS and AD groups, with the probabilities corresponding to each value of the final score as regards its association with “Normality.” The complement to this probability, therefore, should be understood as the “Probability of developing AD.” These two categories will define the two groups to be compared: those defined as normality are labelled as having no dementia MCI—i.e., individuals with a high probability of nondegenerative MCI (nodMCI)—while those linked to the probability of developing AD are labelled as having preclinical AD (preAD).

The initial screening of individuals with nodMCI formed a group of 73 people (20 men and 53 women; 85% of the total were older people, with an average age of 78.73 years and 8.78 years of schooling), and in turn, the group of people with a probable preclinical state of AD (preAD) consisted of 13 older people (four men and nine women; 15% of the total, with an average age of 82.92 years and 8.31 years of schooling). The two groups (see Table 1, Mann-Whitney *U* value and probability) were equivalent in terms of age (*P* = 0.28), years of schooling (*P* = 0.29), and gender (*χ*^2^ test = 0.62, *P* = 0.803). They were also equivalent in terms of cognitive mental state evaluated with the MMSE (*P* = 0.283), in the semantic verbal fluency tests evaluated with the Isaacs Test (*P* = 0.733), in the phonological verbal fluency tests (*P* = 0.765), in the overall value of the MIS scale (*P* = 0.181), and in the Boston Naming Test (*P* = 0.15). We did not find any differences, either, in emotional state measured by the Goldberg anxiety and depression scale (*P* = 0.788). The only differences we found between the two groups corresponded to the values of the Buschke MIS Free Retrieval Test (*P* < 0.04), with those defined as preAD recording less free retrieval of words.

### 2.2. Instruments

Audio recordings were made in a soundproof room with a noise level < 35 dB and a reverberation time of less than one second, using a portable wave recorder and a head-mounted condenser microphone (MiC Plus from Apogee) placed about 14 cm from the speaker's mouth. The microphone was a dynamic unidirectional cardioid, with a frequency range of 20 Hz-20 kHz, sensitivity of 2.5 mV/Pa (-52 dBV), and impedance of 600 ohms. Only the vowel nucleus was used for the acoustic analysis, which was conducted manually. Each sound was edited, using the phonation nucleus and discarding each sample's attack.

### 2.3. Procedure

This study is part of a broader one on AD screening in nursing homes within a permanent collaboration agreement with the State Reference Center for Alzheimer's where the tests are performed. The project has received the approval of the Ethics Committee at the State Reference Centre for Alzheimer's of Salamanca (Spain), a center belonging to the Ministry of Social Affairs.

The neurocognitive evaluation was carried out in three one-hour sessions led by a professional psychologist from a center specializing in the assessment of dementia. The recording sessions lasted around 40–60 min and included a participant interview and speech recordings. Our research method involves individuals reading the first paragraph of “Don Quixote” by Miguel de Cervantes (see appendix). The paragraph, in modern Spanish, contains 126 syllables. The text was displayed on a computer screen in 48 font size to make the reading easier. Importantly, even though the text is not phonetically balanced, it was specifically chosen because the first sentence “En un lugar de la Mancha…” (In a village in La Mancha…) was very familiar to all the participants. This was not the case with the second sentence, which called for strained fluency. We focus on the study of the more sensitive temporal parameters [43] involved in the speech rhythm [32]. Recordings were made in mono at a sampling rate of 44.1 kHz at 16-bit amplitude quantization. Each recording was analyzed using Praat software (version 6.0). Praat determines pitch using acoustic periodicity detection on the basis of autocorrelation—the correlation of a time-domain signal with itself [44]. This technique is more accurate, noise-resistant, and robust than alternative methods, such as those based on cepstrum or combs. A pitch floor of 75 Hz and a pitch ceiling of 300 Hz for men and 100-500 Hz for women with a Hanning window length of 0.01 s were used in accordance with the programmers' recommendations [44].

### 2.4. Statistical Analyses

The speech parameters used between older people with nondegenerative MCI and those expected to develop AD were compared using a Mann-Whitney *U* test of independent samples given the difference in case size between the two groups.

## 3. Results

Table 2 shows the differences between nodMCI and preAD groups in each parameter and the statistic and associated probability values for each one of them. The cases in which the difference between the two groups is significant are marked with asterisks. The statistical analysis reveals clear perceptual differences between the preAD and nodMCI groups in different parameters.

In speech fluency and regarding the parameters of duration, we find significant differences between the two groups in reading time (*P* = 0.005) and phonation time (*P* = 0.002), with the duration being longer for the preAD group. As regards this study's main focus—disruptions in rhythm—it enables us to identify the alterations in language fluency while analyzing the duration of vowel and consonant intervals and the standard deviation in those durations. As regards speech fluency, we do not find any differences in either articulation ratio (*P* = 0.592) or speech ratio (*P* = 0.191). However, we can confirm that preAD individuals record more pauses (*P* = 0.021). As regards the rhythm parameters, we find significant differences between the two groups in the number of syllabic intervals (*P* = 0.002), with more syllabic intervals for the preAD group and more standard deviations in the duration of syllabic intervals (Syll_Interv_*Δ*Standar, *P* = 0.006). An analysis of speech rhythm reveals differences in the nPVI (*P* = 0.002), with higher rhythmic variability in individuals with preAD than in those with nodMCI. We do not find any differences either for average duration of syllabic intervals (Syll_Interv_DAverage, *P* = 0.481) or the coefficient of variation in the duration of syllabic intervals (Syll_Interv_DVarco, *P* = 0.130).

As regards the fundamental frequency parameters, there are no differences between the two groups in fundamental frequency (F0, *P* = 0.634). This parameter may be determined by gender differences, confirming that women use higher frequencies than men (*F*_2,81_ = 11.109, *P* < 0.01). We checked to see whether there are any differences between the two groups depending on gender, and we did not find any differences either among men or among women. As regards the parameters of spectral analysis, the values of asymmetry (*P* = 0.04, parameter of VAD-AD) showed a significant negative skew with a concentration of energy in low frequencies in individuals with a high probability of developing dementia, with the opposite skew in those that do not appear to be developing it. There are no differences between the two groups in the values of the frequencies of the center of gravity (CoG_Hz, *P* = 0.06), but there are differences between the two experimental groups in the standard deviation of the degree of diffusion in the spectrum regarding its center of gravity, with the latter being higher among people at risk of dementia (CoG_sd, *P* = 0.02). A voice quality index that analyzes the adjustment of sound intensity to the frequency values of formants and their respective energies is the one measured by means of the long-term average spectrum (LTAS). We find significant differences between the two groups, with a lower intensity in the overall spectrum of frequencies in individuals with probable preAD (Ltas_mean, *P* = 0.001), with their mean standard deviation also being lower (Ltas_stdesv, *P* = 0.001). The differences in intensity between the two groups appear significantly in the low-frequency range between 50 and 1000 Hz (*P* = 0.008). The differences in intensity appear in women (Ltas_mean, *P* = 0.001; Ltas_stdesv, *P* = 0.003; low-frequency range between 50 and 1000 Hz, *P* = 0.009), but not in men (Ltas_mean, *P* = 0.13; Ltas_stdesv, *P* = 0.21; low-frequency range between 50 and 1000 Hz, *P* = 0.43). This effect does not appear in high frequencies above 1000 Hz.

As regards the parameters of intensity, we do find differences in the value in decibels of the mean amplitude (intensity dB mean, *P* = 0.002), with a lower mean intensity being used by the preAD group. When we consider gender, there are significant differences among women (*P* = 0.001), but not among men. We did not find any differences in the standard deviation of amplitude (intensity dB_sd, *P* = 0.98). We did not find any differences in the VAD-AD parameters Amplit Difference Max Mean (*P* = 0.24) and Amplit Minim (*P* = 0.36). Neither of the two records any differences between the groups analyzed separately in Amplit Difference Max Mean men (*P* = 0.68) or women (0.25) nor in Amplit Minim men (0.68) or women (0.25).

Finally, regarding the overall parameters of voice quality, we do not find any differences between the two groups in the standard values of vocal noise, such as the harmonics-to-noise ratio (HNR, 0.281) or in the dysphonia values evaluated by the Acoustic Voice Quality Index (AVQI, *P* = 0.551). However, as we can see in these values, both groups provide clinical evidence of dysphonia, with the value being above six (the cutoff score between 0 normophonia and 10 dysphonia is somewhere around three [45]). There are no differences, either, in the parameters that make up the AVQI, jitter values (local, *P* = 0.519), shimmer (Loc_dB, 0.199), and voice breaks (%, *P* = 0.088).

## 4. Discussion

This study has once again revealed the difficulties involved in the use of neuropsychological tests for the presymptomatic diagnosis of AD, given that screening tests do not show significant differences in the various scales between those individuals with a speech phonotype close to AD (preAD) and those without those speech characteristics (nodMCI). In both groups, when the elders request a cognitive assessment, they score an average of 23/24 points in the MMSE, which places them in the MCI group. A small proportion of the people within this group would be expected to suffer from an insidious, progressive but slow cognitive decline, a period that may last from three to six years depending on several factors such as age, cardiovascular health status, or cognitive reserve. In contrast, the rest of the MCI would be predicted not to show progressive cognitive deterioration. Only the MIS test's score for free retrieval showed differences between the two groups, leading us and many other scholars to suggest the importance of short-term memory tasks without prompts for predicting AD [38].

It is noticeable that, in our study, the nodMCI group is much larger in size than the preAD group. Beyond the aesthetic pleasure derived from studies comparing two groups of equal size, there is nothing unusual or inaccurate on unequal sample sizes per se. In our design, the belonging to each group cannot be randomly allocated. The sample size imbalance is due to the design. A quasiexperimental design by definition lacks random assignment, i.e., studies in which the allocation of participants to condition is predetermined rather than manipulated by the researcher [46]. This is always the case for ex-postimpact evaluation designs, for example, diagnostic restrictions [47]. Therefore, the size of our groups is proportional to the size that each of them represents on the total population. The preAD group is expected to be smaller (15%) than that of people with nodMCI (85%).

It could be argued that the difference in size between these two groups could affect their heterogeneity, thus compromising the results, i. e., variations in age, gender, or social background. Nevertheless, that has not been the case in our study. A major effort was made to match the participants in terms of any important characteristic that might affect performance in speech [48]. It is important to note that age, gender, and mental and affective state did not show differences between both groups in the preclinical period of the disease.

However, when we tested the homoscedasticity of the two samples, we found a significant value in Levene's test regarding age (*F* = 10.945, *P* < 0.001), a key factor in the screening of AD. It should be noted that, with a small sample, the data displayed on a histogram may be skewed. If we are forced to use a small sample size, we might also be forced to use a nonparametric test. In order to minimize that possible effect (which could also be corrected through Levene's test), it seems prudent to use nonparametric tests, such as the Mann-Whitney *U* test. This test is used to compare two independent groups when dependent variables are continuous. The Mann-Whitney *U* test is the nonparametric equivalent to the two-sample *t*-test. While the *t*-test makes assumptions about a population (i.e., that the sample came from a normally distributed population), the Mann-Whitney *U* test makes no such assumption. The Mann-Whitney *U* test has several advantages, including more statistical power when the assumptions of equality of variances and normality have been violated; when the assumptions have not been violated, they can be almost as powerful as the parametric ones; and small sample sizes are acceptable as is the case of our study [49].

Different sample sizes and the use of nonparametric tests are common in studies with people with dementia. Voice studies like López-De-Ipiña et al. [50], Hoffmann et al. [23], or Beltrami et al. [51] have followed this path.

As regards the 12 parameters used in the study that have proven to be significant for distinguishing between those people with MCI that may or may not develop AD, only two of the parameters of the VAD-AD recorded differences between the two groups: skewness (asymmetry in the spectral feature) and nPVI (variability in articulatory rhythm). This confirms the difference between the language impairments that characterize AD compared to the parameters present in the disorder's preclinical stages. In addition, it justifies the evolution in speech throughout the course of the disease.

This study has focused on the extent to which the metrics of speech rhythm can be used as parameters for predicting the future development of dementia in individuals with MCI and whether these results can help to explain the language processes involved in the early impairment of AD. The results have shown that subtle changes affect the duration of phonation, speech fluency, the spectral distribution of speech, and even the average intensity of speech. As regards duration of phonation, individuals with preAD take longer to read the same paragraph, with more phonation time and more pauses.

The values of the rhythm metrics revealed a number of significant differences between the groups, with the preAD sample vocalizing more syllabic intervals that also have more temporal variability. However, the preAD recording a lower speech periodicity or isochrony in the values of mean duration between successive normalized syllabic pairs (nPVI), in which the standard deviation value considers the syllabic ratio and the number of intervals. These data seem to define individuals with preAD as having more imprecise utterances and stammering more, with more speech pauses in the syntactic boundaries, greater variability in the number of syllabic boundaries with high rhythmic variability, and lower periodicity and isochrony. The presence of these characteristics would be related to developing dementia in the future. By contrast, it appears that the increase in speech time is not due to a slowing of the articulation and speech rate, but instead to changes in speech fluency. The specific neuromechanical pathways would not reveal any impairment in the early stages of cognitive decline.

As regards the parameters of the fundamental frequency of older people, they do not seem to be altered in these initial stages of the disorder's onset. The lack of differences does not depend on gender. As regards spectral analysis, those individuals at greater risk of dementia do record alterations in their voices' spectrographic intensity. They concentrate their energy on higher frequencies, with greater variability and diffusion of their center of gravity, whereas those individuals that will not develop dementia have a greater skewness with a tendency toward low frequencies. Nevertheless, a more specific value such as LTAS recorded a difference between the two groups in voice intensity, and less variability in mean intensity. This effect is especially apparent in the low-frequency spectrum and in women. The frequencies below 1000 Hz pertaining to formant 1 and these results seem to evoke a strained phonation [52] at low frequencies. However, the few cases of men with a probability of dementia detract from this gender difference.

As regards the intensity of the sound, we find that people with a greater probability of dementia express themselves with a lower mean intensity than people that will not develop dementia. The difference is significant in the female gender; women lose more voice intensity. We did not find other alterations in speech intensity. Regarding voice quality, we have found that both groups clearly record similar values of dysphonia, typical of voice changes during the aging process. Neither do we find any differences between them in general voice quality measured by the AVQI or in other specific values such as Jitter, shimmer, and HNR.

These findings have implications for the use of acoustic metrics when characterizing speech performance in the early stages of dementia. Older people with MCI take many years to develop clear neuropsychological symptoms of a future onset of dementia. These individuals have a subjective sense of loss of memory and other impairments in other areas of their lives, but these are not dysfunctional. This research shows that the presence of certain specific rhythmic features in speech performance differentiates these individuals, revealing subtle problems that cannot be perceptively identified when planning the speech that distinguishes individuals with MCI that are more or less likely to develop AD. We have found that these features are not related to older people's tone, acoustic and phonological measures, etc. In most cases, they are features of phonation speed, duration, and intensity. The preAD group is characterized by inappropriate pausing behavior, low speech volume and changes to fluency, monotonous intonation, and in some cases, a lower speech tempo. However, not all of these are necessarily perceived as disordered rhythm. Instead, such deficits are primarily associated with the changes in speech timing and the poor coordination between articulatory systems experienced by speakers with neurogenic speech disorders [53]. Finally, the general set of parameters we have obtained related to the probability of developing dementia seems to confirm the relationship between preclinical dementia and parameters of duration, spectral features, and articulatory rhythm. Obtaining a “high probability of dementia” profile, together with the existence of a cognitive deficit noted in neuropsychological tests, could be a key indicator of the presence of dementia, which must be confirmed by means of a biomarker.

The confirmation of this study's findings will necessarily involve verifying the clinical expressions of the individuals with MCI that have taken part in this study, requiring their longitudinal monitoring to track their future evolution and make an intrasubject comparison of their voice samples [8]. Regarding the assignment of the participants to any of the groups, it has been done according to an algorithm that provides the probability of developing AD via speech analysis. When this algorithm is applied to the speech samples of people with MCI, as it not a heterogeneous group, two different subgroups can be established depending on their degree of similarity either to the speech of people with nonpathological senesce or to that of people with AD. Therefore, the prognosis in this article is only based on a probabilistic model, and the confirmation of this study's findings will necessarily involve verifying the clinical expressions of the individuals with MCI that have taken part in this study, requiring their longitudinal monitoring to track their future evolution and make an intrasubject comparison of their voice samples [8]. As future work, it would be interesting to clarify the role of specific neurological dysfunctions on the alterations of speech.

## Figures and Tables

**Table 1:** Groups' characteristic mean (SD). Mann-Whitney *U* test (standardised test).

	nodMCI group (*n* = 73)	preAD group (*n* = 13)	Mann-Whitney *U* (standardised test)
Average age	78.73 (10.07)	82.92 (5.02)	385 (-1.080)
Men-women	20 (27%)–53 (73%)	4 (31%)–9 (69%)	
Years of schooling	8.78 (4.13)	8.31 (4.71)	558 (1.048)
MMSE ponderated	23.25 (4.63)	24.31 (4.37)	380 (-1.073)
Buschke' MIS total	4.96 (2.04)	4.11 (1.36)	315 (1.339)
Buschke' MIS FreeRetr	1.22 (1.21)	0.33 (0.50)	346 (2.019)^∗^
Buschke' MIS CuedRetr	1.83 (1.18)	2.44 (1.01)	282 (0.812)
Boston Denomination Test	11.02 (4.61)	7.50 (5.89)	81 (-1.455)
Fluency Isaacs' Test	31.52 (7.14)	32.69 (5.75)	434 (-0.341)
Phonological fluency	6.53 (3.03)	6.62 (3.30)	437 (-0.299)
Goldberg's Depression Test	4.73 (4.45)	4 (3.11)	456 (0.269)

^∗^ *P* < 0.05.

**Table 2:** Comparison of the voice parameters used between older people with nondegenerative MCI and those expected to develop AD. Mann-Whitney *U* test of independent samples and means of the two groups.

Parameters	nodMCI mean (SD)	preAD mean (SD)	Mann-Whitney *U* (standardised test)
Duration parameters			
Duration (reading time)	47.48 (22.20)	61.74 (20.95)	243 (-2.791)^∗∗^
Phonation time (sec)	32.35 (10.39)	39.44 (8.75)	213 (-3.153)^∗∗^
Speech rate	3.35 (0.80)	3.07 (0.92)	583 (1.308)
Pauses number	25.79 (16.34)	34.23 (14.64)	283 (-2.310)^∗^
Speech fluency and rhythm parameters			
Normalized_PVI	54.27 (5.42)	59.97 (5.76)	215 (-3.128)^∗∗^
Syll_Interv_DAverage	0.19 (0.03)	0.20 (0.02)	416 (-0.705)
Syll_Interv_*Δ*Standar	0.10 (0.02)	0.11 (0.02)	246 (-2.755)^∗∗^
VARCO of syllabic interval duration	0.51 (0.07)	0.55 (0.09)	349 (-1.513)
Syllabus interval number	154.01 (51.88)	178.08 (33.94)	223 (-3.033)^∗∗^
Articulation rate	4.63 (0.59)	4.54 (0.60)	519 (0.537)
Fundamental frequency and spectral analysis			
F0 mean (Hz)	168.61 (28.90)	164.82 (20.21)	514 (0.476)
F0 mean (Hz_men)	138.86 (25.17)	138.84 (16.31)	41 (0.077)
F0 mean (Hz_women)	172.12 (16.34)	170.09 (13.73)	250 (0.230)
Asymmetry (skewness, Hz)	11.33 (4.14)	8.62 (4.38)	645 (2.056)^∗^
Center of gravity (CoG_Hz)	446 (98)	568 (227)	321 (-1.851)
Center of gravity SD (CoG_sd)	547 (191)	753 (318)	276 (-2.393)^∗^
LTAS mean (dB)	32.46 (2.31)	30.20 (2.00)	763 (3.478)^∗∗^
LTAS mean (dB_men)	32.41 (2.64)	30.47 (2.33)	30 (-0.878)
LTAS mean (dB_women)	32.48 (2.20)	30.07 (1.97)	401 (3.247)^∗∗^
LTAS SD (dB)	40.32 (2.10)	38.16 (2.82)	738 (3,177)^∗∗^
LTAS SD (dB_men)	40.21 (2.31)	38.46 (3.08)	57 (1.317)
LTAS SD (dB_women)	40.37 (2.03)	38.03 (2.88)	388 (2.987)^∗∗^
LTAS range 50-1000 Hz	44.87 (2.03)	43.26 (2.37)	694 (2.646)^∗∗^
LTAS range 50-1000 Hz_men	46.24 (2.12)	44.76 (3.27)	51 (0.852)
LTAS range 50-1000 Hz_women	46.40 (2.06)	43.98 (4.12)	369 (2.608)^∗∗^
LTAS 1 kHz-2 kHz	27.13 (3.29)	26.21 (4.98)	513 (0.464)
LTAS 2 kHz-4 kHz	17.87 (4.59)	17.94 (2.98)	480 (0.066)
Intensity parameters			
Intensity mean (dB)	75.36 (1.77)	73.63 (2.00)	735 (3.141)^∗∗^
Intensity mean (dB_men)	74.94 (0.41)	73.94 (0.91)	55 (1.162)
Intensity mean (dB_women)	75.98 (0.41)	73.51 (0.60)	390 (3.027)^∗∗^
Intensity mean_SD (dB)	17.28 (10.54)	14.77 (2.94)	551 (0.922)
Intensity SD (dB_men)	19.24 (14.31)	12.76 (4.26)	51 (0.852)
Intensity SD (dB_women)	16.55 (8.78)	15.66 (1.79)	198 (-0.809)
Amplitude minimum	-9.08 (13.53)	25.01 (12.44)	551 (0.922)
Intensity Minim (dB_men)	-23.19 (129.34)	27.59 (14.61)	46 (0.465)
Intensity Minim (dB_women)	-3.76 (107.83)	23.85 (12.14)	285 (0.929)
Intens Diferenc Max-Min Mean (dB)	95.55 (13.40)	61.76 (12.26)	377 (-1.175)
Intens Dif Max-Min (dB_men)	109.22 (129.34)	58.79 (14.07)	34 (-0.465)
Intens Dif Max-Min (dB_women)	90.37 (107.68)	63.07 (12.03)	181 (-1.149)
Acoustic Voice Quality parameters			
Jitter (local)	2.42 (1.07)	2.35 (0.66)	427 (-0.567)
Shimmer Loc (dB)	1.12 (0.27)	1.19 (0.22)	394 (-0.965)
Voice breaks (%)	38.93 (11.01)	43.93 (13.24)	333 (-1.706)
Acoustic Voice Quality Index (AVQI)	6.00 (1.18)	6.17 (0.95)	425 (-0.597)
HNR (dB)	12.96 (3.50)	11.91 (3.18)	570 (1.151)

^∗^ *P* < 0.05; ^∗∗^*P* < 0.01; ^∗∗∗^*P* < 0.001.

## Data Availability

The data matrix of this study can be consulted if requested by the editor. The matrix data used to support the findings of this study are restricted by the the Unit for the Promotion of healthy aging and prevention of cognitive problems associated with the aging process of the University of Salamanca in order to protect PATIENT PRIVACY. Data are available from Juan José García Meilán, meilan@usal.es for researchers who meet the criteria for access to confidential data.

## Appendix

### A. Original Version

In a village of La Mancha, the name of which I have no desire to call to mind, there lived not long since one of those gentlemen that keep a lance in the lance-rack, an old buckler, a lean hack, and a greyhound for coursing. An olla of rather more beef than mutton, a salad on most nights, scraps on Saturdays, lentils on Fridays, and a pigeon or so extra on Sundays, made away with three-quarters of his income.

### B. Translated Version

En un lugar de la Mancha, de cuyo nombre no quiero acordarme, no ha mucho tiempo que vivía un hidalgo de los de lanza en astillero, adarga antigua, rocín flaco y galgo corredor. Una olla de algo más vaca que carnero, salpicón las más noches, duelos y quebrantos los sábados, lantejas los viernes, algún palomino de añadidura los domingos, consumían las tres partes de su hacienda.
